# Ubiquitination‐Driven Reprogramming of Proteostasis in Metastasis

**DOI:** 10.1002/advs.202522165

**Published:** 2026-01-20

**Authors:** Dongping Wei, Jiayan Chen, Yaping Xu

**Affiliations:** ^1^ Medical Research Center The First Affiliated Hospital of Wenzhou Medical University Wenzhou Zhejiang China; ^2^ Department of Radiation Oncology Fudan University Shanghai Cancer Center Shanghai China; ^3^ Department of Radiation Oncology Shanghai Pulmonary Hospital School of Medicine Tongji University Shanghai China

**Keywords:** DCAF12, metastasis, proteostasis, TRiC/CCT, ubiquitination

## Abstract

Metastasis, the leading cause of cancer‐related mortality, poses a fundamental proteostatic challenge, requiring rapid and precise proteome remodeling in response to stress. While ubiquitination is linked to protein degradation, our recent work uncovered a non‐canonical, metastasis‐promoting mechanism centered on DCAF12, a substrate receptor of the Cullin 4–RING ubiquitin ligase complex. DCAF12 mediates non‐degradative ubiquitination of TRiC/CCT chaperonin subunits, allosterically activating the chaperonin to enhance its assembly, stability, and folding capacity. This ubiquitination‐dependent activation circuit enables metastatic cells to efficiently fold and stabilize diverse pro‐metastatic proteins, thereby facilitating dynamic proteome reprogramming. Herein, we present the DCAF12–TRiC/CCT axis as a central regulatory component of this adaptive response, explore its evolutionary basis, and propose DCAF12 as a prototype for a broader class of “DCAFome” regulators of chaperone function. This mechanistic understanding establishes a direct rationale for therapeutically targeting this axis to disrupt adaptive proteostasis. Moreover, we outline a therapeutic paradigm termed “proteostatic stress creation.” This framework encompasses a spectrum of strategies, from precision protein–protein interaction inhibitors to state‐selective degraders of DCAF12 or its ubiquitinated chaperonin subunits. These approaches can potentially disrupt the DCAF12–TRiC/CCT axis, thereby undermining the proteostatic resilience that sustains advanced cancers.

## Graphical abstract

The DCAF12–TRiC/CCT axis is a key regulator of metastasis in cancer. By reprogramming proteostasis to ensure efficient protein folding, it drives progression through a dual mechanism: enhancing cancer cell motility and invasiveness while concurrently activating pro‐growth and survival pathways. This synergy between enhanced cellular movement and sustained pro‐tumor signaling facilitates robust and efficient metastatic spread.

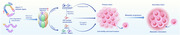

## 1 Non‐Degradative Ubiquitination Directs Metastatic Adaptation

Cancer metastasis is a complex, multistep process that encompasses tumor cell detachment, local invasion, intravasation, circulatory survival, extravasation, and colonization of distant tissues [1, 2]. Progression through these stages requires tumor cells to continuously adapt to fluctuating microenvironments and dynamically reprogram their behavior [2, 3]. While transcriptional reprogramming initiates these adaptations, their rapid execution critically depends on the proteostasis network, which maintains a delicate balance among protein synthesis, folding, trafficking, and degradation to meet stage‐specific proteostatic demands [3, 4, 5, 6].

Molecular chaperones and the ubiquitin‐proteasome system (UPS) are the central pillars of proteostatic regulation [7, 8]. Conventional models often oversimplify the interplay between these two systems, portraying the proteasome primarily as a disposal unit—a passive downstream effector of chaperone activity for misfolded proteins [8, 9]. However, this degradation‐centric view has recently been revised based on the growing consensus that ubiquitination is a versatile and multifaceted post‐translational modification, directly modulating protein activity, stability, and interactions through diverse chain topologies, thereby functioning as a potent signaling mechanism [10, 11, 12]. Our recent study identified a regulatory axis involving DCAF12, a substrate receptor within the Cullin 4–RING (CRL4) ubiquitin ligase complex [13]. We found that DCAF12 mediates the non‐degradative ubiquitination of all eight subunits of the TRiC/CCT chaperonin complex through K63‐ or K27‐linked chains, a modification that enhances both chaperonin stability and client‐folding capacity [13]. This finding establishes ubiquitination as an allosteric activator of chaperone function, thereby expanding its functional spectrum beyond degradation and positioning it as a direct driver of proteostatic adaptation during metastasis. This facilitative role is mechanistically grounded in the diverse array of TRiC/CCT clients, including cytoskeletal proteins essential for motility, cell cycle regulators critical for proliferation, and oncogenic signaling molecules [13, 14, 15]. By co‐opting the DCAF12–TRiC/CCT axis, cancer cells establish a master regulatory node that simultaneously amplifies multiple metastatic capabilities [13]. Therefore, ubiquitination‐dependent proteostasis regulation forms an essential post‐translational layer that integrates with transcriptional reprogramming to equip tumor cells with dynamic plasticity essential for metastasis.

## 2 From DCAF12 to the DCAFome: An Expansive Chaperone Regulatory Network

**FIGURE 1:**
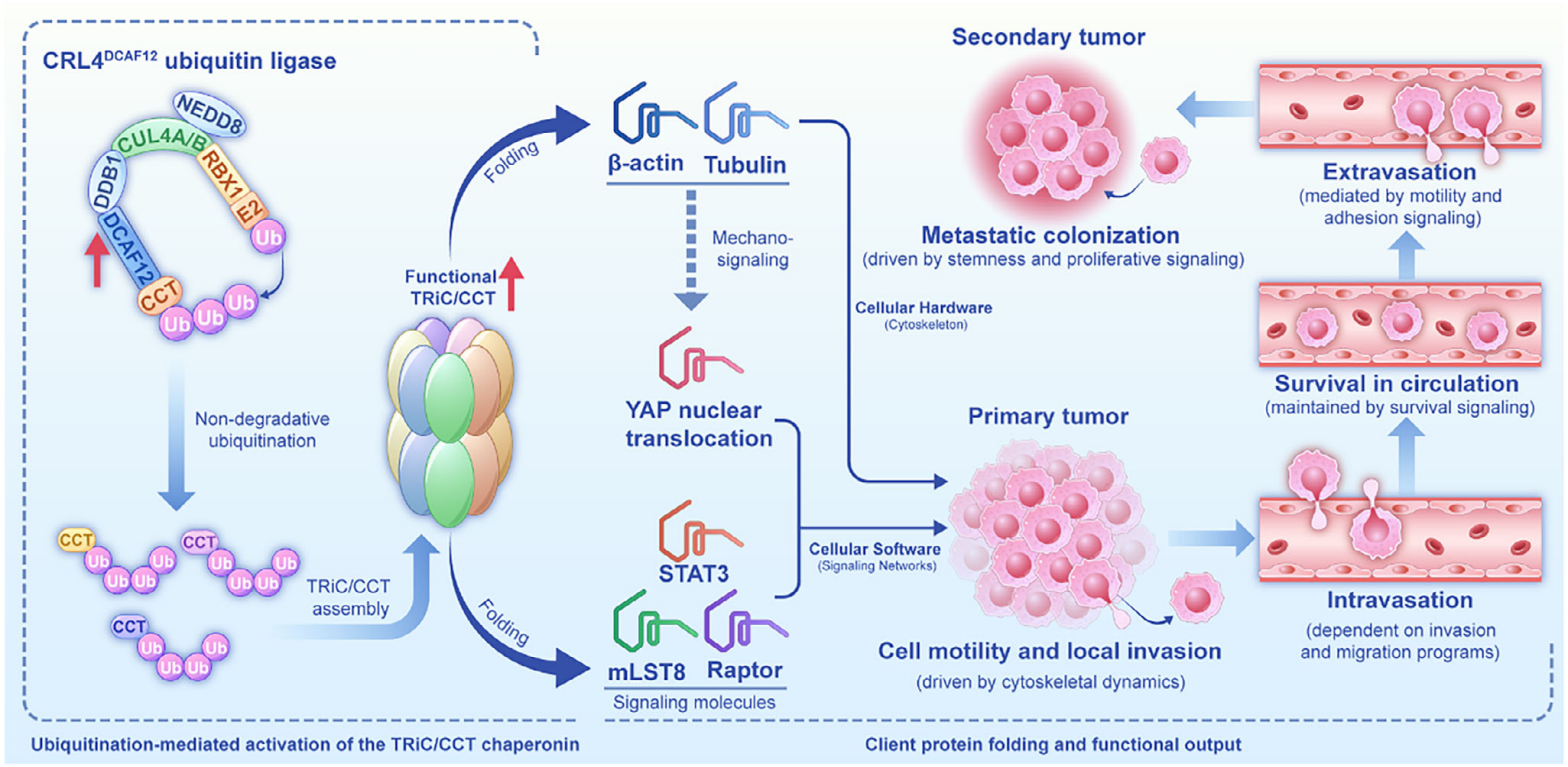
Central Regulatory Axis for Metastatic Proteostasis The DCAF12–TRiC/CCT axis functions as a critical integrative hub in metastasis by coordinating the folding of cytoskeletal components (e.g., β‐actin and tubulin)—which enables force generation and mechanosignaling—and oncogenic signaling “software” (e.g., STAT3, Raptor, and mLST8). As previously demonstrated [13], CRL4‐DCAF12 ubiquitin ligase mediates non‐degradative ubiquitination of TRiC/CCT subunits, promoting their assembly into a hyper‐assembled state with enhanced folding capacity. This activation enables the synchronized maturation and stabilization of functional pro‐metastatic clients, thereby promoting both motility/invasion and activation of key proliferation, survival, and stemness pathways, including YAP, STAT3, and mTOR. By co‐opting proteostatic control, this axis acts as a master regulatory node that drives multiple stages of the metastatic cascade.

The discovery of the DCAF12–TRiC/CCT axis establishes a new paradigm, revealing an integrated mechanism that coordinates the mechanical and signaling infrastructure essential for cancer metastasis. DCAF12‐mediated ubiquitination enhances the folding of structural proteins (e.g., β‐actin and tubulin) to promote force generation, mechanosignaling, cell motility, and invasion, while concurrently promoting the maturation of key signaling molecules (e.g., STAT3, Raptor, and mLST8). By activating pro‐metastatic pathways, such as STAT3, YAP, and mTOR, this axis drives proliferation, survival, and stemness [16, 17, 18], which are essential for metastatic progression. This dual regulation of cytoskeletal “hardware” and signaling “software” positions the axis as a central integrator of metastatic competence in cancer cells (Figure 1). The anti‐metastatic effects observed upon disruption confirm the indispensable role of this signaling axis [13].

Notably, this axis illustrates evolutionary repurposing. In *Drosophila*, DCAF12 acts as a ubiquitin‐independent tumor suppressor that promotes apoptosis [19]. However, in mammals, it has been repurposed as a ubiquitination‐dependent oncogenic driver. This functional shift likely reflects the increased complexity of mammalian tissue homeostasis, wherein stress response pathways are co‐opted to fuel malignancy. Given that the WD40 β‐propeller structure is a defining feature of the DCAF family [20, 21, 22], we propose that the regulatory mechanism uncovered for DCAF12 is not an isolated phenomenon. Therefore, we nominate DCAF12 as the founding member of a specialized class of chaperone regulators and call for a re‐evaluation of the DCAF protein family. Of the approximately 90 DCAF proteins encoded by the human genome [23], most remain poorly characterized. We suggest that diverse DCAF proteins may employ analogous mechanisms to regulate distinct chaperone systems, including HSP90 and HSP70. A conceptual precedent for this model exists in the E3 ligase CHIP, which dynamically regulates the chaperone network through mechanisms, such as substrate‐dependent feedback and competitive complex formation [24, 25]. Building on this precedent, we hypothesize that DCAF proteins constitute a dedicated and expansive regulatory network, thereby enabling tumor cells to exert precise, ubiquitination‐dependent control over their proteostasis networks. The validation of this model would significantly expand the functional repertoire of CRL4 ubiquitin ligases.

**FIGURE 2:**
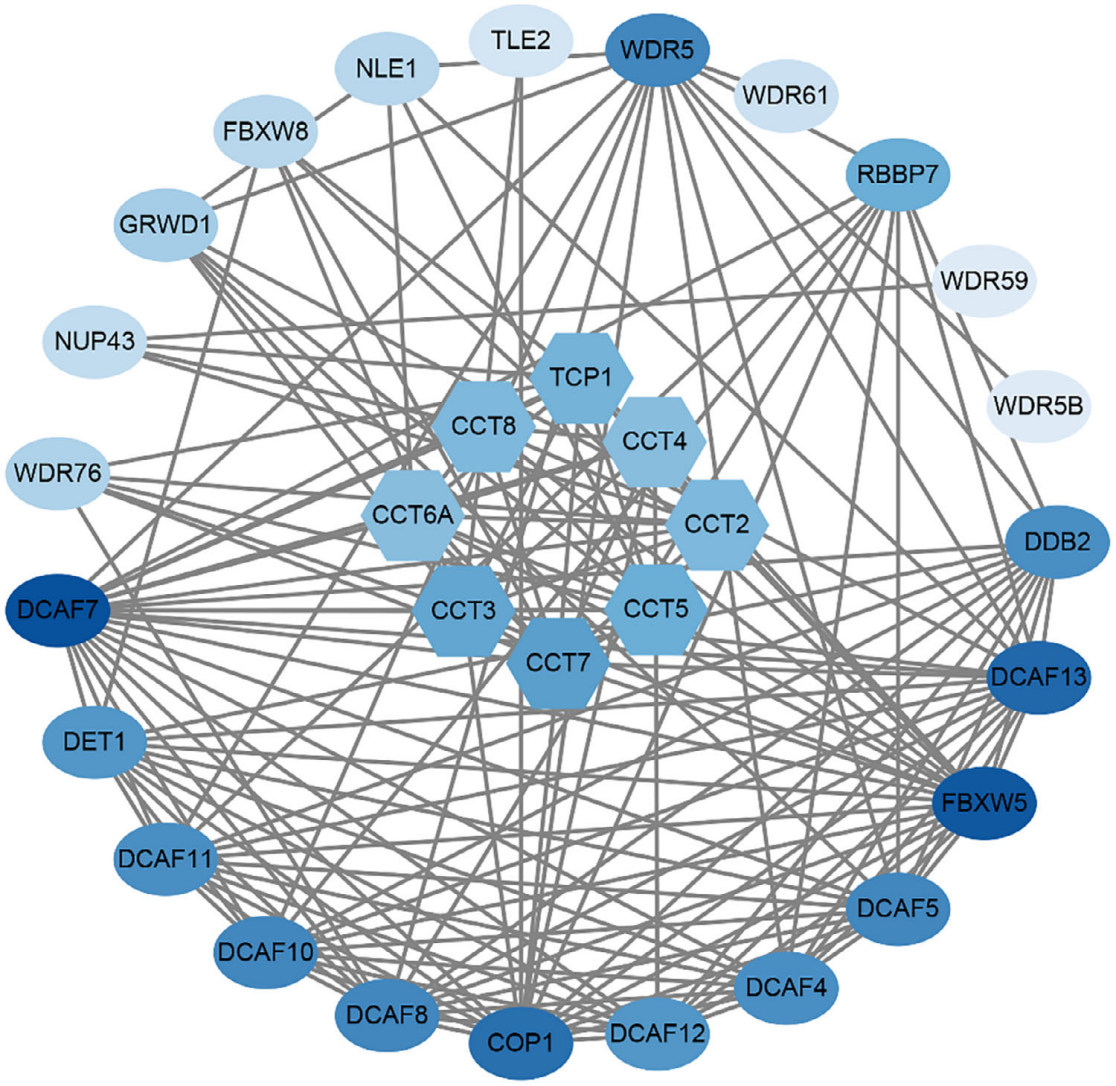
DCAFome: An Expansive Network of Chaperone Regulation The protein–protein interaction network (curated from BioGRID and visualized using Cytoscape) reveals extensive high‐confidence interactions between DCAF family members (circles) and TRiC/CCT subunits (hexagons), demonstrating a regulatory interface that extends beyond the established DCAF12–TRiC/CCT axis. The node color intensity corresponds to the degree of connectivity (darker blue indicates higher connectivity), suggesting that highly connected proteins may play a more central role in the underlying biological processes.

This hypothesis predicts frequent physical interactions between DCAF proteins and the core chaperone machinery, a prediction strongly supported by large‐scale protein–protein interaction data. Specifically, publicly available datasets (e.g., BioGRID: https://thebiogrid.org), which aggregate results from multiple independent proteomic studies, consistently report physical interactions between various DCAF proteins (all sharing a common WD40 β‐propeller scaffold) and different subunits of the TRiC/CCT chaperonin complex [26, 27, 28, 29, 30]. These interactions, primarily revealed by affinity purification‐mass spectrometry, constitute a pervasive network that provides compelling independent evidence of widespread connectivity between DCAF proteins and the core proteostasis machinery. This connectivity is structurally rationalized by the intrinsic ability of TRiC/CCT to recognize WD40 domains common to the DCAF family [20, 21, 22, 31, 32]. The convergence of public interactomic and structural evidence indicates that the DCAF12–TRiC/CCT axis represents a broader regulatory paradigm in cancer. We propose a model of multilayered circuitry in which DCAF proteins interact with TRiC/CCT in three principal capacities: as clients that require TRiC/CCT for their own folding, as direct regulators that control TRiC/CCT assembly or activity through ubiquitination (e.g., DCAF12), and as quality‐control effectors that ubiquitinate misfolded substrates retained within the chaperonin chamber. Together, these interactions constitute an integrated regulatory network. This extensive and specific interface between the DCAF family and the chaperone system leads us to conceptualize a “DCAFome”(Figure 2)—an ensemble of DCAF proteins dedicated to ubiquitination‐dependent proteostasis regulation. Within this framework, chaperonins are subject to multiplexed and context‐dependent control, whereby distinct DCAFome members fine‐tune chaperone activity by modifying specific subunits, targeting distinct functional states, or removing aberrant folding intermediates, ultimately enabling the dynamic and adaptive reprogramming of cellular proteostasis.

To elucidate the molecular basis of this fine‐tuning, we propose a working hypothesis for the structural mechanism of DCAF12‐mediated activation, informed by our functional studies showing that DCAF12‐mediated ubiquitination enhances TRiC/CCT assembly [13]. The conjugation of K63‐ or K27‐linked ubiquitin chains to specific CCT subunits may promote assembly through two complementary allosteric mechanisms. First, ubiquitin chains can act as molecular crosslinks, bridging adjacent subunits within or between the two TRiC/CCT rings. This would reduce conformational flexibility, stabilizing the complex into a more rigid, folding‐competent state—a model consistent with the increased complex stability observed upon DCAF12 activation. Second, ubiquitination near critical apical substrate‐binding sites may modulate the dynamics of flexible “lid” segments, facilitating client protein capture, retention, or release during ATP‐dependent folding cycles. Such a mechanism could account for the higher folding efficiency of pro‐metastatic clients in cancer cells. Notably, DCAF12 depletion does not completely abolish TRiC/CCT assembly [13], implying that its ubiquitination function refines complex formation, rather than fundamentally enabling it. In cancer, this refinement is co‐opted and amplified to generate a pathological “hyper‐assembly” state that is critical for metastasis. These models yield testable predictions, particularly that ubiquitinated TRiC/CCT complexes exhibit distinct architectural and dynamic properties. Systematic validation using advanced structural biology techniques, such as cryo‐electron microscopy (cryo‐EM), represents a critical next step.

This regulatory plasticity provides a mechanistic basis for dynamic rewiring of the proteome during metastasis. Collectively, our findings reveal a refined regulatory topology in which the DCAFome constitutes an integrated network of chaperone modulators (Figure 2), enabling cancer cells to dynamically adapt their protein folding capacity to meet the relentless demands of the tumor microenvironment. This expanded view—transforming DCAF proteins from mere substrate receptors to architects of a dedicated chaperone regulatory network—establishes the DCAFome as a key conceptual framework for understanding proteostatic adaptation. Additionally, it provides a mechanistic foundation for therapeutic strategies aimed at exploiting proteostatic dependencies in metastatic cancer.

## 3 Targeting Proteostatic Vulnerabilities to Control Metastasis

The discovery of the DCAF12–TRiC/CCT axis as a central regulator of metastasis opens novel therapeutic avenues with significant clinical implications. Pharmacological inhibition of this axis using HSF1A, a first‐in‐class inhibitor that selectively targets the TCP1 and CCT3 subunits [33], suppresses metastasis with minimal acute toxicity [13], providing essential proof of concept. However, this success reveals a fundamental challenge in targeting core proteostasis—the difficulty in achieving sufficient selectivity to spare normal tissues. Therefore, overcoming this challenge requires innovative strategies.

To define the basis for a potential therapeutic window, we performed functional and genetic analyses in lung cancer cells. Genetic ablation of DCAF12 impaired, but did not completely inactivate, TRiC/CCT activity [13], suggesting that chaperonin function exists on a tunable continuum rather than as a binary on–off switch, consistent with our assumption. Notably, aberrant DCAF12 overexpression in lung cancer cells increases the dependency on TRiC/CCT by promoting ubiquitination of its subunits, leading to enhanced complex assembly and ultimately increased chaperone activity [13]. This ubiquitination‐driven “hyper‐assembly” state is characterized by increased stability of the TRiC/CCT complex and higher efficiency in folding oncogenic clients, such as actin, tubulin, and STAT3, thereby creating a feed‐forward loop that fuels metastatic progression. The therapeutic potential of targeting this axis is further supported by in vivo data showing that systemic DCAF12 ablation in mice is tolerated, permitting normal development without embryonic lethality [34]. Although specific impairments (e.g., in spermatogenesis and immunity) occur due to the stabilization of the novel substrate MOV10 [34], these findings are instructive. They demonstrate that systemic DCAF12 inhibition can be tolerated overall but reveal specific functional vulnerabilities, thereby raising the critical question of where the tolerable limits lie and what mechanisms define them. These observations collectively inspired the concept of “proteostatic stress creation”, a strategy to selectively induce proteostatic crises within metastatic cells. This paradigm exploits the intrinsic vulnerability of advanced cancer cells, whose dependence on DCAF12‐driven chaperonin activity forces them to operate near the limit of their proteostatic capacity. Consequently, their proteostasis network is poised at a precarious threshold, where even minor perturbations can trigger a cascade of misfolding and functional decline that normal cells, with their robust buffering capacity, can withstand. Therefore, even partial inhibition of this axis, achievable with agents, such as HSF1A, can elicit potent anti‐metastatic responses while sparing normal cells from toxicity.

However, a thorough understanding of the mechanism‐based toxicities is required to translate this “proteostatic stress creation” paradigm into safe and effective therapies. Beyond degrading MOV10 [34], DCAF12 is thought to be involved in maintaining genomic stability through the degradation of its key substrate, MCMBP, which regulates DNA replication [35]. Consequently, DCAF12 inhibition may disrupt replication fidelity, posing an additional on‐target risk to rapidly dividing normal tissues, such as the bone marrow and intestinal epithelium. While the link between MOV10 stabilization and specific tissue toxicities provides an initial safety signal [34], the mechanistic underpinnings of how DCAF12 loss‐of‐function translates to pathophysiology remain undefined. Therefore, by regulating both proteostasis and genomic stability [13, 35], DCAF12 sharpens the definition of the therapeutic index. This frames the central challenge of calibrating treatments that selectively stress metastatic cells (which are hyper‐dependent on DCAF12‐mediated TRiC/CCT assembly [13]) while minimizing the impact on normal proliferative tissues that rely on it for DNA replication and other specialized functions [35]. This dual nature underscores that the therapeutic window is not merely a function of differential expression but rather a precise balance between pathogenic hyperactivity and essential basal functions. Therefore, the next critical step is to determine whether on‐target toxicities arise primarily from the cumulative stabilization of key substrates, disruption of specific functional modules of DCAF12, or compensatory rewiring of dependent cellular networks. To advance this strategy, a focused preclinical agenda is required to map these interconnected toxicities and develop tailored mitigation approaches, such as intermittent dosing. Successfully navigating this integrated safety profile is essential to validate the core premise that metastatic cells can be selectively eliminated through calibrated proteostatic stress. Ultimately, this line of investigation will determine the translational potential of targeting proteostatic vulnerabilities as a viable strategy for controlling metastasis.

## 4 Precision Therapeutic Strategies for Metastatic Control

**FIGURE 3:**
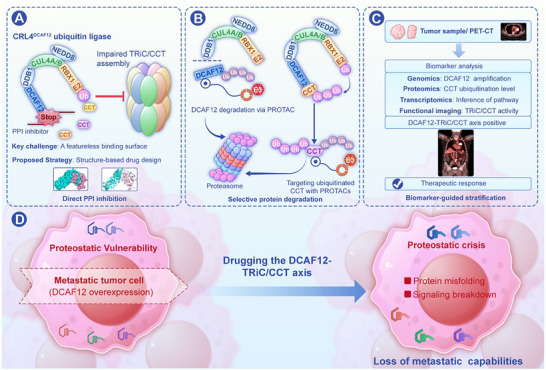
Therapeutic Targeting of Proteostatic Vulnerability These schematic outlines strategies to trigger a selective proteostatic crisis in metastasis‐competent cells by targeting the DCAF12–TRiC/CCT axis. The model integrates three complementary, biomarker‐informed approaches: (A) direct disruption of the TRiC/CCT complex using PPI inhibitors that block the DCAF12–TRiC/CCT interface, thereby suppressing complex formation. (B) Catalytic removal of axis components using PROTAC‐based degraders, including targeting either DCAF12 or ubiquitinated TRiC/CCT subunits (e.g., CCT5). This method impedes the hyper‐assembled chaperonin pool essential for metastasis. (C) Biomarker‐guided patient stratification to identify tumors reliant on DCAF12–TRiC/CCT activity using markers, such as DCAF12 amplification, subunit ubiquitination signatures, or downstream pathway activation. The efficacy of these interventions (D) is underpinned by their ability to exploit the differential proteostatic capacities of normal and malignant cells. While normal cells possess a robust proteostatic buffering capacity that enables them to tolerate such perturbations, metastasis‐competent cells, operating near their functional limits due to oncogenic addiction to the DCAF12–TRiC/CCT axis, are selectively driven into a terminal proteostatic crisis.

Building on the mechanistic insights into the DCAF12–TRiC/CCT axis, we outline a multifaceted therapeutic framework designed to exploit the unique proteostatic vulnerabilities of metastatic cells. This framework rests on three pillars (Figure 3): (1) Precision interference through direct inhibition of the DCAF12–TRiC/CCT protein–protein interaction; (2) Catalytic removal of key axis components through proteolysis‐targeting chimeras (PROTACs); and (3) Patient stratification guided by a theranostic biomarker framework to ensure that the right therapy is matched to the right patient.

### 4.1 Precision Interference

As the first pillar of this framework, pharmacological disruption of the DCAF12–TRiC/CCT interaction offers a paradigm shift from “global inhibition” to “precision interference.” Genetic knockout or broad catalytic inhibition of DCAF12 stabilizes substrates, such as MOV10 and MCMBP, which likely underpins the observed toxicities in spermatogenesis, immunity, and proliferative tissues [34, 35]. In contrast, a molecule that specifically blocks the DCAF12‐TRiC/CCT interaction could decouple the pro‐metastatic “hyper‐assembly” of TRiC/CCT driven by DCAF12's ubiquitination function. This strategy acts as a highly selective molecular intervention targeting pathological reinforcement within cancer cells while sparing baseline protein functions. Our previous proof‐of‐concept study using engineered peptide inhibitors supports the feasibility of this approach, demonstrating that targeted disruption of this PPI suppresses metastasis without compromising basal cell viability, a phenotype distinct from the cytotoxicity induced by depletion of core TRiC/CCT subunits [13].

The central translational challenge in advancing this “precision interference” strategy is to evolve peptide‐based probes into orally available small‐molecule inhibitors, overcoming the well‐known hurdles of drugging PPIs [36]. Although the DCAF12–CCT5 interface elucidated by high‐resolution cryo‐EM studies has emerged as a starting point [37, 38], the broader interaction landscape between DCAF12 and the remaining TRiC/CCT subunits remains unclear. Therefore, we lack a comprehensive structural view of this dynamic, multipartite interface, which likely exhibits classic yet daunting PPI features, such as a large, flat, and dynamic surface with sparse and shallow pockets [39]. To bridge this gap, we propose an integrated discovery strategy that begins by employing molecular dynamics simulations on available and new structural data to capture interface dynamics, identifying transient pockets and hotspot residues [40, 41, 42]. These insights feed into a dynamic pharmacophore model, guiding a fragment‐based drug design campaign. Initial fragments are then optimized through structure‐guided linking or growing to simultaneously engage multiple hotspots, improving affinity toward lead‐like properties [43, 44]. A key insight enabling this strategy is that the pervasive interaction between DCAF12 and multiple subunits of the TRiC/CCT complex creates inherent target redundancy, thereby amplifying the vulnerabilities available for pharmacological intervention. Our earlier findings suggest that impairing DCAF12 binding to any single TRiC/CCT subunit, as demonstrated for CCT3, CCT5, and CCT7 [13], may be sufficient to disrupt the chaperonin's pro‐metastatic assembly and client folding activity. This redundancy not only broadens the chemical space for inhibitor discovery but may also impose a higher evolutionary barrier to resistance development. The need for cancer cells to simultaneously overcome inhibition at multiple redundant interfaces (e.g., DCAF12–CCT3, –CCT5, and –CCT7) to achieve full resistance presents a significantly less probable evolutionary path than circumventing a single targeted interaction. Therefore, targeting this multipartite interface not only expands long‐term therapeutic opportunities against this axis but also increases the probability of developing durable treatment strategies.

### 4.2 Catalytic Removal

To achieve more profound and sustained therapeutic effects, we move beyond occupancy‐driven inhibition to a strategy of catalytic removal. This approach employs PROTACs, which are bifunctional molecules designed to eliminate, rather than merely inhibit, the key components of the DCAF12–TRiC/CCT axis. Leveraging the advancements and clinical validation of PROTAC technology [45, 46, 47], this approach offers a potent and translatable therapeutic paradigm by harnessing the cell's degradation machinery. Within this framework, two distinct degrader modalities emerge as particularly compelling, each exploiting a unique vulnerability node within the axis: (i) Direct degradation of DCAF12 to ablate the upstream regulator. This strategy is significantly bolstered by the recent clarification of the DCAF12 WD40 domain structure and its substrate‐binding pocket, which selectively identifies C‐terminal di‐glutamate degrons [38]. This interaction presents a promising target for designing a PROTAC ligand or “warhead.” Although this approach is technically feasible, it carries the risk of on‐target toxicity due to DCAF12's pleiotropic roles in CRL4‐mediated substrate recognition beyond the TRiC/CCT axis [34, 35]. (ii) Precision, state‐selective degradation of downstream effectors, specifically the ubiquitinated forms of TRiC/CCT subunits (e.g., CCT3 or CCT5) that preferentially interact with DCAF12 [13]. This method exploits the endogenous ubiquitination mark to selectively degrade only ubiquitinated TRiC/CCT subunits, thereby inhibiting the pathological “hyper‐assembly” of chaperonin complexes engaged in metastasis‐promoting client folding, while preserving the essential baseline pools required to maintain global proteostasis [48]. Therefore, this “state‐selective” degradation paradigm promises to achieve a superior therapeutic index by directly dismantling the oncogenic signal while preserving physiological function. While direct DCAF12 degradation offers a broad upstream attack, state‐selective degradation of ubiquitinated TRiC/CCT subunits represents a more precise strategy to cripple the pathological axis with potentially fewer systemic effects. The case of DCAF12‐ubiquitinated CCT5 exemplifies both the elegant rationale and formidable inherent challenge of this approach. The mechanistic basis lies in assembly‐dependent degron exposure, where only monomeric or assembly‐disrupted CCT5 (a state induced or marked by DCAF12‐mediated ubiquitination) exposes its C‐terminal degron for recognition. However, its success hinges on achieving exquisite ligand selectivity for the ubiquitinated state over its unmodified counterpart. Notably, ubiquitination can significantly alter the substrate conformation and surface properties [49, 50, 51, 52, 53, 54]. Although this phenomenon is increasingly recognized, its full implications for ligand design warrant further exploration. This remodeling tends to produce transient “conformational signatures” as opposed to classic deep pockets [49, 50, 51, 52, 53, 54], presenting an underexplored, yet promising design paradigm. Consequently, targeting these features necessitates innovative ligand discovery strategies that embrace methods capable of capturing transient conformational states, probing allosteric surfaces, or identifying dynamic protein‐ubiquitin interfaces.

To translate this paradigm into a viable therapy and overcome the fundamental selectivity hurdle, we propose two complementary, structure‐informed design pathways that eschew brute‐force empirical screening in favor of a mechanistic approach. These approaches treat the ubiquitinated state as a distinct molecular entity using mechanistic insights to guide precise ligand designs: (i) Structure‐based targeting of the ubiquitination‐induced state. This design capitalizes on two principal—and often intertwined—consequences of ubiquitination: the creation of a novel composite epitope at the ubiquitin‐protein interface and conformational remodeling that may expose cryptic binding sites. The workflow starts with high‐resolution structural elucidation of the ubiquitinated TRiC/CCT subunit (e.g., via cryo‐EM) to reveal a static composite interface. This is followed by molecular dynamics simulations to map ubiquitination‐induced dynamics and identify transiently exposed, ligandable pockets. These insights enable structure‐guided virtual screening or fragment‐based design to develop complementary binders, such as small molecules, cyclic peptides, or proteomimetics, which specifically recognize the altered surface topography of the modified protein. (ii) Engineering bivalent PROTACs for avidity‐driven selectivity. This strategy enhances the fundamental logic of PROTACs by designing molecules capable of simultaneous and cooperative engagement with the target protein and its covalently attached ubiquitin. Such a degrader achieves high functional avidity only when both epitopes are present and correctly positioned specifically on the ubiquitinated TRiC/CCT subunit. This “dual‐point recognition” mechanism combines avidity with catalytic elimination, establishing a robust selectivity filter imposed by the precise spatial and stoichiometric constraints of the substrate binding. Together, these rational design pathways constitute an emerging strategy in post‐translational modification–guided drug design.

### 4.3 Patient Stratification

Beyond the development of therapeutic modalities, clinical translation hinges on a theranostic biomarker framework capable of identifying tumors functionally dependent on the DCAF12–TRiC/CCT axis and guiding the selection of the optimal therapeutic strategy. This demands a shift from static biomarkers to a dynamic, multi‐tiered system that aligns biomarker signatures with specific therapeutic vulnerabilities, effectively matching the appropriate therapeutic modality to the specific molecular weakness of each tumor. We propose a three‐tiered biomarker stratification system. The first (upstream dependency) tier encompasses genomic alterations (e.g., *DCAF12* amplification) or transcriptional signatures that indicate chaperonin hyper‐demand, identifying patients likely to respond to upstream disruption through PPI inhibitors or DCAF12 degraders. The second (core mechanistic) tier captures pathway activation through the direct detection of DCAF12‐mediated ubiquitination of specific TRiC/CCT subunits, identifying optimal candidates for state‐selective degraders. The third tier (functional consequences) assesses downstream proteostatic stress, as evidenced by client protein stabilization (e.g., MOV10) or integrated stress response activation. Tumors in this tier, operating near the threshold of proteostatic failure, are predicted to be exquisitely sensitive to precision interference or rational combination therapy.

To translate this mechanistic framework into clinical practice, a parallel focus on biomarker validation is as critical as therapeutic development. Although this stratification logic is mechanistically grounded, its clinical validity remains to be established. Bridging this gap requires a rigorous, multi‐phase validation framework. The first phase involves retrospective analyses of well‐annotated cohorts using techniques, such as quantitative immunohistochemistry, targeted mass spectrometry, and RNA sequencing. These studies not only correlate biomarker presence with metastatic progression but also explore differential outcomes across biomarker subtypes to preliminarily test the predictive logic of stratification. Simultaneously, the development and standardization of robust, clinical‐grade assays (e.g., companion diagnostics) are needed. The ultimate validation will come from prospective clinical trials in which patient enrollment and treatment assignment are explicitly guided by this biomarker tier, definitively proving that matching therapy to the biomarker profile maximizes therapeutic benefit.

The integration of functional diagnostics, such as imaging probes capable of dynamically monitoring TRiC/CCT assembly or activity in vivo, represents a technically challenging but potentially informative direction. Such tools could enable the real‐time identification of tumors operating nearest their proteostatic limit, offering a dynamic readout of therapeutic susceptibility and ultimately enabling the strategic administration of the right intervention at the time of maximal tumor vulnerability.

## 5 Future Horizons: Key Questions and Technological Frontiers

The DCAF12–TRiC/CCT axis likely exerts a broader influence within the tumor microenvironment (TME). Palm et al. revealed that TRiC/CCT directly regulates lysosomal acidification by binding to and stabilizing the V1 domain (the ATP‐hydrolytic domain) of the V‐ATPase proton pump [55]. Despite the open question of whether this effect depends on TRiC/CCT's chaperone activity, the discovery positions the DCAF12–TRiC/CCT axis as a potential upstream regulator of tumor acidosis, a hallmark of cancer progression [56]. Therefore, we hypothesize that the aberrant activation of this axis enhances V‐ATPase V1 domain stability, thereby increasing functional proton pump delivery to the plasma membrane. Given the dual localization of V‐ATPases on lysosomal and plasma membranes [57, 58, 59]—with the plasma membrane pool being instrumental in the extracellular acidification exploited by cancer cells [57, 58]—this axis constitutes a critical determinant of extracellular pH. By driving the acidification of the tumor niche, this axis may facilitate key pro‐tumoral processes, including immune evasion and matrix remodeling, ultimately fostering therapy resistance [56].

However, validating this hypothesis and delineating the axis's full functional scope face three major, interconnected challenges. First, its mechanistic workings at atomic scale remain opaque. Although TRiC/CCT subunit ubiquitination has been documented [13], the precise structural and allosteric consequences of DCAF12‐mediated non‐degradative ubiquitination—particularly its impact on chaperonin assembly, activity, and client specificity—are largely unresolved. Future breakthroughs will likely require high‐resolution cryo‐EM structures of ubiquitinated chaperonin complexes to move our understanding from correlation to causation. Second, tools for dynamic, functional observation are lacking. Connecting molecular events to microenvironmental phenotypes demands the ability to monitor the axis's functional output in real time within living systems. The development of advanced biosensors capable of tracking protein folding, complex assembly, and localization in live cells will be essential to empirically define how this axis dynamically regulates V‐ATPase function and pH homeostasis. Third, a critical gap persists in validating these findings in physiologically relevant contexts. The ultimate assessment of any therapeutic potential must employ models that faithfully recapitulate native tumor–microenvironment interactions. Advanced metastasis models that preserve this complexity will serve as crucial proving grounds for testing whether targeting this axis can achieve sustained metastatic suppression through microenvironmental reprogramming.

Given the novel and potentially context‐specific nature of DCAF12‐mediated non‐degradative ubiquitination of TRiC/CCT subunits, as suggested by our findings [13], future studies must prioritize orthogonal validation to establish a robust mechanistic framework. Verifying DCAF12‐mediated non‐degradative ubiquitination and subsequent TRiC/CCT activation is critical and requires independent biochemical replicates. Analysis of public proteomic or genomic datasets could uncover correlations between the expression, interaction, or mutational patterns of key axis components and patient outcomes or TME features, providing independent corroboration of our findings. Ultimately, confirmation through collaboration with independent laboratories would represent the gold standard for ensuring result reproducibility. Collectively, these orthogonal approaches will transform our hypothesis from a conceptual model into a mechanistically validated and therapeutically actionable target.

Extending from this specific pathway, the DCAF12–TRiC/CCT paradigm illustrates a broader principle: ubiquitination can serve as a precise “tuner” of chaperone networks. This insight suggests the existence of a wider “DCAFome” regulatory layer over proteostasis, potentially extending its reach to other chaperone systems (e.g., HSP90/HSP70), which are known to be modulated by ubiquitination [60]. Deciphering this code, which involves understanding how tumors dynamically rewire their proteostasis networks through dedicated E3 ligases, constitutes a fundamental challenge with profound therapeutic implications. Consequently, the DCAF protein family emerges not only as a collection of substrate receptors but also as a sophisticated regulatory network that confers adaptive proteostatic plasticity to cancer cells. This plasticity, an evolutionary strategy to withstand diverse microenvironmental and therapeutic stressors, not only underpins metastatic resilience but also represents a compelling new class of therapeutic vulnerabilities.

Translating these insights into clinical practice will demand a coordinated, multidisciplinary effort that mirrors the biological complexity involved. Mechanistically, it is essential to delineate how cellular stress signaling dynamically modulates DCAF12 function across distinct stages of metastatic progression. Translationally, the rational development of small‐molecule inhibitors must advance in parallel with the identification of predictive biomarkers through comprehensive molecular profiling of tumors reliant on this axis. Given the intrinsic buffering and redundancy within proteostasis networks, durable clinical efficacy will likely require rational combination therapies that co‐target the DCAF12–TRiC/CCT axis and complementary proteostatic nodes to induce synthetic lethality. Ultimately, clinical success will hinge on integrating deep mechanistic understanding, cutting‐edge structural insights, and robust translational models to achieve durable metastasis suppression through the targeted disruption of the adaptive processes that sustain it.

## 6 Conclusion: Rewiring Proteostasis—From Mechanism to Therapy

This Perspective identifies DCAF12‐mediated non‐degradative ubiquitination as a direct allosteric activator of the TRiC/CCT chaperonin, advancing our understanding of proteostatic regulation in cancer. This mechanism provides a rapid post‐translational strategy for metastatic adaptation, enabling proteome remodeling independently of de novo transcription. By coordinately enhancing the folding of diverse pro‐metastatic client proteins, the DCAF12–TRiC/CCT axis serves as a pivotal accelerator for the execution of metastatic programs, effectively bridging the gap between adaptive signaling and structural implementation.

Our findings suggest a paradigm shift in ubiquitin signaling, extending its role beyond that of a degradation tag to an allosteric regulator of chaperone activity. This functional specialization renders metastatic cells dependent on augmented and dynamically rewired folding capacity, thereby revealing a core vulnerability. This discovery not only underpins the concept of “proteostatic stress creation” but also unveils multiple therapeutic avenues directly targeting this weakness, including direct disruption of the DCAF12–TRiC/CCT interaction, selective degradation of ubiquitinated subunits, and biomarker‐guided patient stratification. Collectively, these approaches represent direct translational extensions of core mechanistic findings, highlighting a clear link between mechanism and therapy.

At a broader level, this Perspective reinforces the significance of the DCAFome by positioning ubiquitin‐mediated chaperone regulation as an emerging principle of proteostatic control in cancer. The structural logic of WD40 domain interactions implies that the DCAFome represents a more extensive regulatory network, with the DCAF12–TRiC/CCT axis as a prototype. Future studies should delineate the full scope of this network, define its functions within the tumor microenvironment, and develop selective agents to disrupt its dysregulated components. This focus on targeting the proteostatic adaptability that enables metastasis, rather than individual oncoproteins, shifts the therapeutic paradigm from a reactive pursuit of moving targets to a proactive dismantling of the cancer's adaptive foundation. We anticipate that deciphering this interplay will catalyze a new generation of therapies designed to suppress metastasis by exploiting its proteostatic vulnerabilities, thereby transforming the treatment of advanced cancers. Ultimately, rewiring the proteostatic networks that metastatic cells depend on for survival could provide a central therapeutic leverage point to their sustained suppression.

## Conflicts of Interest

The authors declare no conflicts of interest.

## Data Availability

The data that support the findings of this study are available from the corresponding author upon reasonable request.
